# Vitality of the Teeth

**Published:** 1848-01

**Authors:** Wm. B. Ross


### VITALITY OF THE TEETH.

BY WM. B. ROSS, D. D. S.

The connection of the teeth with the general system is unlike any other connection of its parts or appendages; the articulation is strong, and the position of the teeth in the operation of their functions immovable. The substance, situation and articulation of the teeth are in accordance with the innumerable displays of infinite wisdom and goodness exhibited by the Creator in the formation of man. Admirably adapted by their strength and relative position to bear the greatest pressure that the muscles destined to act upon them can possibly exert, while their crowns are covered with one of the hardest substances in nature. Indeed, we can hardly conceive of any substance that would undergo the same amount of wearing with so little wasting.

They are, however, like other portions of sin smitten nature, subject to decay and dissolution; and when affected by disease their extraction becomes in many cases indispensable. In this case the strength and external insensibility of the organ aid in its extirpation. The pain of removing a tooth is generally very great, and were it not that the operation is of short duration, it would indeed be most formidable. It is somewhat remarkable that an organ of such exquisite internal sensibility, and such firm attachment should be removed with so little inconvenience and be followed by consequences of so little importance.

No part of the human body possessing *vitality* could be separated from it with a less amount of pain, or the wound heal with so small an amount of suffering.

The loss of the teeth, then, which becomes so often necessary, may be regarded as a merciful calamity. The reason for this is found in its peculiar organization and that of its investing membrane. The vessels which enter into this membrane and serve in part to give life to the tooth are so small that the blood which issues from them when they are all broken up is in general very inconsiderable. After the removal of a tooth we find that there exists in nature a provision for the absorption of the alveolus by which it had been sustained. From these facts it seems to have been the design of the Creator that teeth, after having become useless and offensive, should be removed.

In what does the vitality of the teeth consist? and by what laws is it governed? are enquiries of no little importance to the Dental and Medical professions. Which acts the most important part in sustaining this vitality, the pulp or the periosteum? It is supposed by some that the pulp is by far the most important agent from the fact that the tubuli extending from the pulp cavity reach nearly to the outer portion of the body of the tooth; and, as a matter of course, the vessels and nerves filling them have the same extensions and exert a corresponding influence. But does it follow that the longest vessels and nerves perform the most important part in sustaining animal life? This question can only be tested by experience when it can be brought to bear upon the case. One fact in relation to this matter which is established by experience and observation is, that teeth often remain in the socket after the nerve is dead and totally annihilated for years, with but little if any change of appearance, and without any inconvenience. The destruction of the periostum of course involves the loss of the tooth, for want of mechanical support; but stripped of this difficulty, would any one having a knowledge of physiology suppose the tooth to suffer as little inconvenience from the loss of the periostum as it is known to do from the loss of the pulp.

Neither the nerve of a tooth nor its investing membrane seem to have much inherent predisposition to disease. The nerve of a tooth is seldom painful, unless exposed by the decay of the tooth; nor is the periostum often diseased unless invaded by a diseased gum at the alveolar border, or by a diseased nerve at the apex of of the fang. They may both partake of general inflammation of the system, but this is generally neither very painful nor of long continuance. Any settled form of disease is generally, if not always, communicated by a disease of the parts connected with them. There seems, however, this difference between them: for ta nerve partially destroyed by disease, nature exerts no escuperative power, while the diseased periostum is often restored. This position, though occupying disputed ground, is nevertheless sussained by facts which, in the scales of common sense, will always outweigh the ablest theories.

In the formation of the teeth, the ivory is deposited in layers from without inwardly, until sufficient space only is left for the pulp cavity; while the enamel and ecmentum are formed from within outwardly. This fact of itself would lead us to suppose that the vessels and nerves from the pulp would reach through the ivory, while those from without would only reach to it. The investigations of anatomists upon this subject have not enabled them to say whether these vessels inasculate or not. We find, however, that the periostum will sustain the root of a tooth, and keep up vitality in that part with which it is connected; while the internal part is wasting with decay. Instances also occur in which the pulp cavity is entirely obliterated and filled with ossific matter, and this, too, where there is no apparent disease.—Nor is this operation of nature confined to persons far advanced in life; it has been known to occur at from thirty to forty years of age.

Teeth that have lost the nerve are often or rather generally found to be diseased at the root, and in all such cases should be extracted; but it by no means follows that because the nerve is dead the tooth should be extracted, for innumerable instances occur in which teeth with healthy nerves are excised, the nerve destroyed mechanically, and yet the fang remain with entire comfort for many years. The chemical destruction of the nerve is almost invariably followed by alveolar disease; its mechanical, or rather surgical destruction is often effected with impunity.

